# Safe and effective implantation and use of vagal nerve stimulation in new-onset refractory status epilepticus in early pregnancy: a case report

**DOI:** 10.3389/fneur.2023.1183080

**Published:** 2023-05-15

**Authors:** Malaika Jindal, Laura Delaj, Joel Winston, Rishu Goel, Sadia Bhatti, Milena Angelova-Chee, Richard Selway, Laura Mantoan Ritter

**Affiliations:** ^1^Faculty of Life Sciences and Medicine, King's College London, London, United Kingdom; ^2^Department of Neurology, East Kent Hospitals University National Health Service (NHS) Foundation Trust, Kent, United Kingdom; ^3^Department of Neurophsyiology, King's College Hospital, London, United Kingdom; ^4^Department of Obstetrics and Gynaecology, East Kent Hospitals University National Health Service (NHS) Foundation Trust, Kent, United Kingdom; ^5^Department of Obstetrics, King's College Hospital, London, United Kingdom; ^6^Intensive Care Unit, King's College Hospital, London, United Kingdom; ^7^Department of Neurosurgery, King's College Hospital, London, United Kingdom; ^8^Department of Neurology, King's College Hospital, London, United Kingdom

**Keywords:** NORSE, VNS, pregnancy, outcomes, status epilepticus

## Abstract

**Introduction:**

The management of new-onset refractory status epilepticus (NORSE) in pregnancy may be complicated by anti-seizure medication (ASM) polytherapy-associated teratogenicity. We aim to demonstrate the safety and efficacy of vagal nerve stimulation (VNS) in a pregnant patient presenting with NORSE.

**Case description:**

A 30-year old female, at 5-weeks' gestation presented with drug-refractory myoclonic status epilepticus, responsive only to high levels of anesthetic agents. The severity of seizures did not allow extubation, and the patient remained ventilated and sedated. VNS was implanted 26 days after seizure onset. The immediate post-operative output was 0.25 mA, which was rapidly titrated up to 0.5 mA the next morning, and to 0.75 mA that afternoon. This was further increased to 1.0 mA on 3rd day post-operation, and to 1.25 mA 7 days post-op. Myoclonic jerks diminished significantly 7 days post-op, allowing extubation. Twenty days after VNS implantation, no myoclonic jerks were observed. There was also a notable neurological improvement including increased alertness and mobility, and ability to obey commands. Drug overdose was subsequently found to be the most likely etiology of her NORSE. An early pregnancy assessment 17 days after VNS implantation showed a normally sited pregnancy, normal fetal heart activity and crown-rump length. The patient remained seizure free, gained functional independence and delivered a premature but otherwise healthy baby at 33 weeks' gestation.

**Conclusion:**

NORSE is challenging to manage, further compounded in pregnancy due to the teratogenicity of ASMs and ASM polytherapy. This is the first case-study to report the safe implantation and use of VNS during the first trimester of pregnancy for the management of NORSE.

## 1. Introduction

Status epilepticus (SE) is a life-threatening neurological emergency with a significant mortality and morbidity. New-onset refractory status epilepticus (NORSE) is “a clinical presentation, not a specific diagnosis, in a patient without active epilepsy or other pre-existing relevant neurological disorder, with new-onset of refractory status epilepticus without a clear acute or active structural, toxic or metabolic cause. This includes patients with viral or autoimmune causes. If no cause is found after extensive evaluation, this is considered “cryptogenic NORSE” or “NORSE of unknown cause”” (1). It represents ~20% of all refractory status epilepticus cases, and although rare, it can be fatal and often leads to poor neurological outcomes (2). NORSE typically presents in young healthy people, with a viral illness-like prodrome (3).

Although several treatments such as anti-seizure medications (ASMs), anesthetic agents, immunosuppressive treatments, and neuromodulation have been suggested, consensus on optimal management is only now emerging. Management is further compounded in pregnant patients due to the teratogenicity of several anti-seizure medications (ASMs). In these patients, neuromodulation with vagal nerve stimulation (VNS) is an attractive option, as it may help avoid the use of ASM polytherapy. The successful use of VNS for NORSE has been documented in previous case reports/series (4–8), however, there are no reports of VNS use in NORSE in pregnancy. This case is the first to report on the safe and effective use of VNS in NORSE in early pregnancy.

## 2. Case description

### 2.1. Initial management

Our patient was a 30 year-old female, 5-weeks pregnant at presentation, with no history of epilepsy or neurological disease. She was brought to her local emergency department (ED) after she was found shaking and unresponsive by her partner. The seizure lasted for more than 1 h, and was aborted by 10 mg diazepam given by ambulance paramedics. Seizures recommenced in ED and were refractory to intravenous (IV) lorazepam (4 mg) and levetiracetam 1 g. She was subsequently intubated in ED and started on a propofol infusion (100 mg/h). CT head and lumbar puncture on admission were both unremarkable. Magnetic resonance imaging (MRI) and Magnetic resonance venogram (MRV) conducted on day two after onset were normal. The day after admission, the propofol infusion was held, which restarted seizure activity. The seizures started as distal limb myoclonic jerks in the upper and lower limbs, and then progressed to stimulus-sensitive (touch and sound), generalized myoclonus. Sedation was recommenced with propofol, fentanyl (200 micrograms/h) and midazolam (10 mg/h), and the levetiracetam dose was increased to 1.5 g BD. Clobazam 5 mg BD was also added. Generalized myoclonus/myoclonic jerks continued while she was on propofol, fentanyl, clobazam, levetiracetam, and midazolam. Obstetric input was sought, and phenytoin (100 mg TDS), lacosamide (100 mg BD), topiramate (100 mg BD) were added, in sequence. She was pulsed with methylprednisolone from day 5 of presentation.

### 2.2. Pre-operative management in our epilepsy center

Seven days after initial presentation, the patient was transferred to our center with ongoing right arm and left leg myoclonic jerks and head movements suggesting ongoing status epilepticus. Two hundred milligram biotin and pyridoxine 50 mg were commenced on arrival.

**Figure 1:**
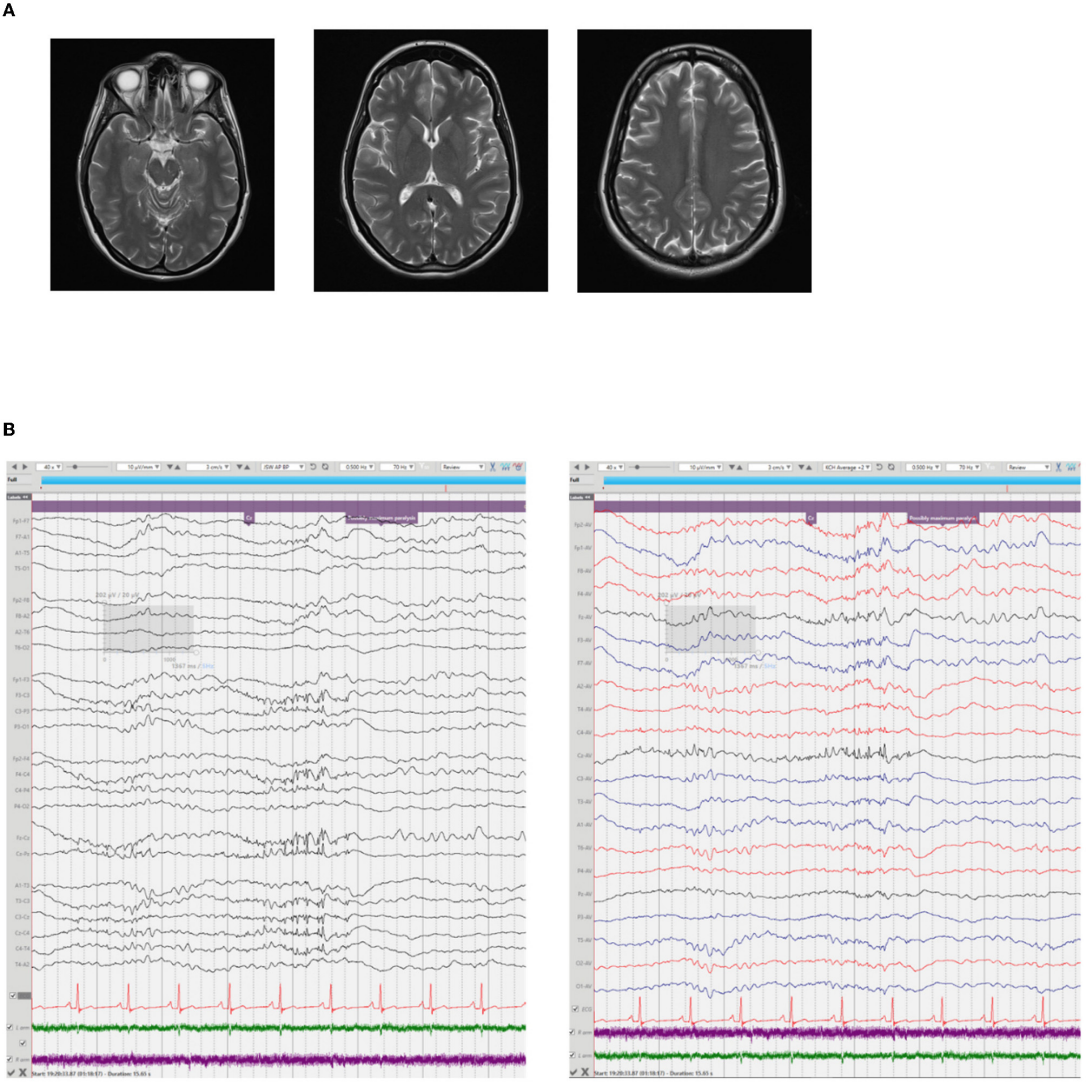
Diagnostic imaging and electroencephalography. **(A)** Magnetic Resonance Imaging of the Brain xx days after onset. T2- weighted axial views at three levels demonstrating normal gray/white matter differentiation, no focal lesions and preserved CSF spaces. **(B)** EEG with muscle relaxant; During the deepest period of paralysis runs of low amplitude spikes are seen at Cz with a fronto-central field in the absence of detectable proximal upper limb EMG or any clinical movements.

The patient's lumbar puncture was repeated on day 7 when she was admitted to our center and she was found to be HSV negative, and aciclovir was stopped. MRI head (Figure 1A) and spine were also repeated (day 11 after onset) and were unremarkable. On admission to our center metabolic, CSF virology, and autoimmune and paraneoplastic panels were all negative (detailed in Table 1), as were QuantiFERON-TB Gold, and blood, CSF and sputum cultures. Vitamin B12, folate and serum ferritin were normal. At this point, a diagnosis of NORSE was suspected, and the patient was managed as such.

**Table 1:** Summary of investigations.

**Investigation**	**Results**
**CSF findings**	
CSF NMDA receptor antibodies	Negative
CSF analysis	Negative
CSF glycine	Normal
CSF oligoclonal bands	Negative
CSF lactate	Raised
CSF virology (including CMV, adenovirus, HSV 1 and 2, EBV, enterovirus and varicella zoster virus)	Negative
**Serum findings**	
Metabolic panel (including Zn, Cu Se, Mg, Ca and phosphate)	Normal
Serum GAD	Negative
Glycine antibodies	Negative
Cytokine innate (including IL1-beta, IL6, TNF-alpha, and IL8)	Normal
NMDA receptor antibodies	Negative
Plasma Acyl Carnitine	Negative
Neuronal antibodies (including Hu, Ri and Yo antibodies)	Negative
Diabetes antibodies (including glutamic acid decarboxylase, IA2 and zinc transporter 8 antibodies)	Negative
Anti-CASPR2 antibodies	Negative
Anti-LGi1 antibodies	Negative
Amphiphysin	Negative
Anti-AMPA-1, AMPA-2 and GABA antibodies	Negative
**Imaging and telemetry findings**	
MRI head	No radiological evidence for explanation of NORSE, no claustrum sign bilaterally, basal ganglia normal bilaterally.
TVS ultrasound (before VNS implantation)	Gestational sac, yolk-sac and embryo present Fetal heart activity present: 171 bpm Both ovaries normal Crown-rump length normal
CT chest abdomen and pelvis	No evidence of malignancy No features of trophoblastic disease or ovarian teratoma
Transabdominal ultrasound (after VNS implantation)	Uterus: anteverted, normal Fetal heart activity present: 169 bpm Crown-rump length: 60 mm Amniotic fluid normal No free fluid Ongoing normally sited pregnancy
EEG on presentation to our center	Included withdrawing sedation and introducing muscle relaxant Interictal EEG showed continuous slow, generalized; at times rhythmic and stimulation induced, background suppression, spikes, central (Cz > C3 = C4, rare spread to Fz/F3/F4/Fp1/Fp2), and eta-delta complexes (rare, seen with stimulation). The patient is still in status epilepticus, by virtue of the existence of midline spikes and myoclonus, both spontaneous and clearly stimulus induced, with clear examples of EMG correlate following central small spikes, confirming a cortically driven process. There is however an improvement as both clinical and EEG changes appear much less prominently after stimulation and interictal midline discharges are less frequent and less prominent.
EEG after VNS implantation	Overall picture appears to be one of stimulus sensitive multifocal myoclonus. There is a clear increase in cortical spiking during the more major attacks and clear correlation of the jerks and spikes suggesting this is cortical rather than of brainstem origin.

The patient also underwent three EEGs: soon after admission to the local hospital CCU, a prolonged study after admission to our center, and after VNS implantation (Table 1; Figure 1B).

Propofol weaning was attempted again, 9 days after onset but this led to increased myoclonic jerks and it was reintroduced. Perampanel 6 mg was commenced via a nasogastric tube 11 days after onset. Five sessions of plasmapheresis (PLEX) were commenced 12 days after presentation. The patient was started on Anakinra (100 mg daily) after the last PLEX. Propofol was cross-tapered with ketamine (2.5 mg/kg/h) to reduce the risk of propofol infusion syndrome as the ketogenic diet as started. The ketogenic diet was commenced 22 days after onset, and Levetiracetam was switched to Brivaracetam (100 mg BD).

An CT chest abdomen and pelvis was performed and ruled out trophoblastic disease or ovarian teratoma.

Whilst there had been no collateral history or evidence of overdose, the toxicology panel conducted on admission to her local hospital, which was made available to us 20 days after onset, demonstrated grossly elevated levels of morphine, fentanyl, pregabalin, and cocaine. Two past admissions for ecstasy and pregabalin overdose also came to light. Based on these results, possible anoxic brain injury was considered although subsequent MRI Brain scans did not show any evidence of hypoxic injury.

Due to refractoriness of her myoclonus on weaning sedation, an unremarkable MRI brain study and difficulty in assessing her level of consciousness off sedation, the Epilepsy Surgery MDT agreed on VNS implantation. VNS was implanted 26 days after seizure onset.

### 2.3. VNS implantation and outcome

#### 2.3.1. Outcome of status epilepticus

**Figure 2:**
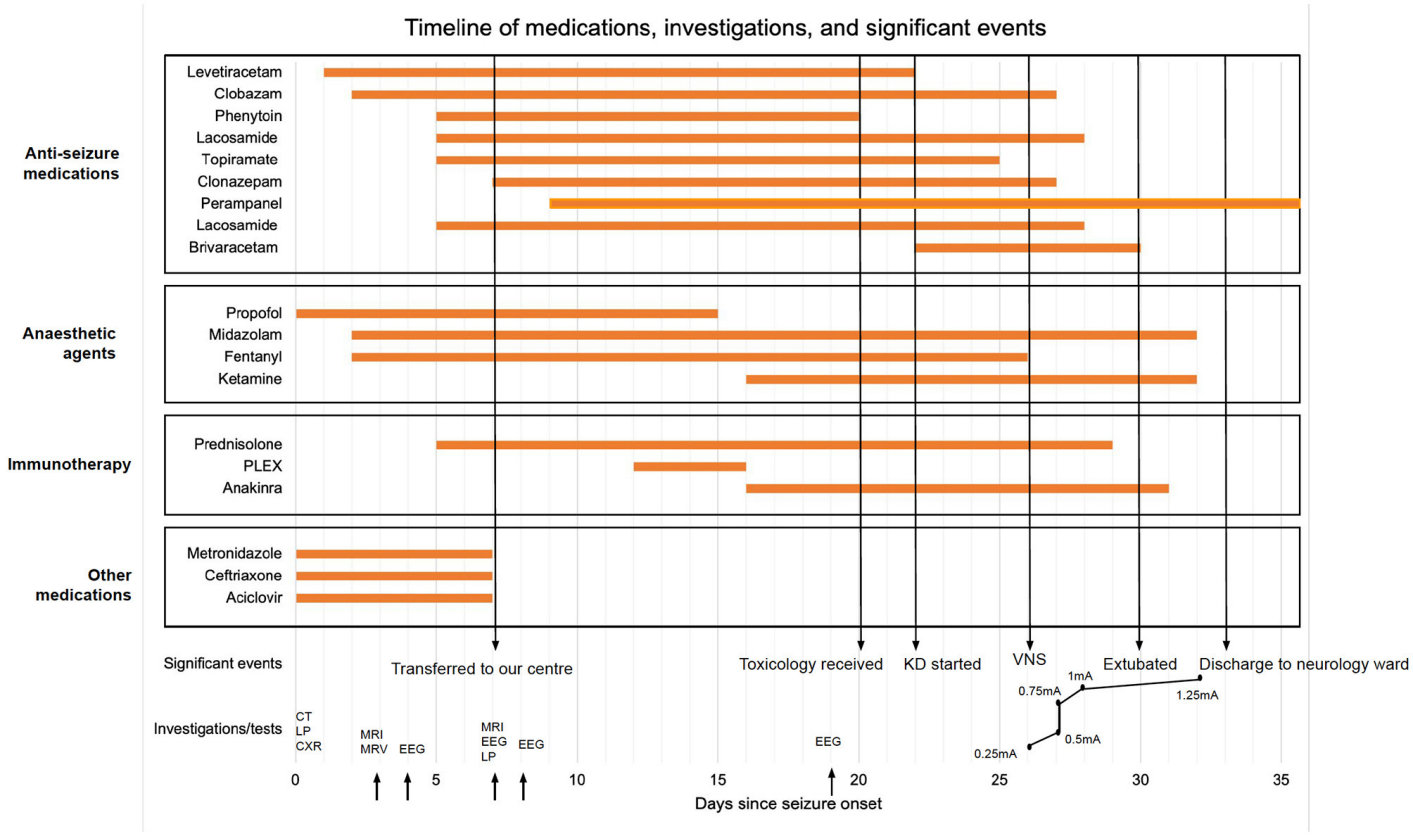
Timeline of care; KD, ketogenic diet; VNS, vagal nerve stimulation; CT, computed tomography; LP, lumbar puncture; CXR, chest x-ray; MRI, magnetic resonance imaging; MRV, magnetic resonance venogram; EEG, electroencephalogram.

The clinical outcomes post-VNS implantation have been outlined in Table 2. The VNS was switched on day 0 with immediate post-operative settings 0.25 mA, 30 Hz, 500 μs, 30 s on, 5 min off, Duty cycle 10%. VNS output was increased till day 7 post-op according to Figure 2. All other parameters stayed the same. No intraoperative or post-operative complications were noted. Two days post-operatively, myoclonic jerks were still occurring, but eye opening to voice and apparent purposeful movements (including reaching for the tube) were noted. On day 3 post-operatively slow ketamine weaning was started. The patient was extubated on day 5 post-op. Semi-purposeful movement in all four limbs was noted, and the stimulus-sensitive myoclonic jerks had diminished significantly. Midazolam was stopped. No myoclonus was noted 7 days post-op. The patient was discharged to a neurology ward in our center. Anakinra, the ketogenic diet, and prednisolone were weaned. Thirteen days after VNS implantation, the patient demonstrated full passive range of motion in all limbs. After 18 days, normal power was noted in all limbs (MRC 4+/5). Nineteen days later the GCS had improved to 15/15. The patient showed comprehensible speech, and was oriented to place and person. Twenty days after VNS implantation, no myoclonic jerks were noted, and the patient was transferred back to her local hospital for further rehabilitation. Four months after VNS implantation, occasional, inconsistent tremors in upper and lower limbs were noted thought to be either a functional overlay or enhanced physiological tremor: these movements were clinically distinctively different from myoclonic jerks, were distractable and could be voluntarily suppressed. A repeat EEG performed at about 5 months from hospital admission did not capture the tremor-like movements; it showed background slowing and occasional spikes with no clinical correlate (no motor manifestations). Five months after VNS implantation, no further seizures or focal neurology were reported. The patient continued to take clonazepam and perampanel and mobilized using a Sara Steady sit-to-stand aid. Fatigue and low mood were reported.

**Table 2:** VNS titration, clinical and electrographic progress.

**Day**	**Event**
Day 26	VNS implantation Post-operative VNS output: 0.25 mA
Day 27	VNS output increased to 0.50 mA VNS output increased to 0.75 mA Myoclonic jerks still occurring Eye opening to voice Apparent purposeful movements
Day 28	VNS output increased to 1.00 mA Slow ketamine weaning started Prolonged EEG: overall picture appears to be one of stimulus sensitive multifocal myoclonus. There is a clear increase in cortical spiking during the more major attacks and clear correlation of the jerks and spikes suggesting this is cortical rather than of brainstem origin.
Day 30	Patient extubated Semi-purposeful movements noted in all limbs Stimulus-sensitive myoclonic jerks diminished significantly Midazolam stopped
Day 32	VNS increased to 1.25 mA No myoclonus noted Patients commenced on folic acid 5 mg OD and vitamin D 10 micrograms OD
Day 33	Patient discharged to neurology ward in our center
Day 38	Full passive range of motion in all limbs
Day 43	Early pregnancy assessment by obstetrics team Fetal heart activity normal Normal crown-rump length Gestational age: 12 weeks + 3 days Concluded that pregnancy was developing normally
Day 44	Normal power in all limbs noted (MRC 4+/5)
Day 45	GCS 15/15 Speaking and comprehensible Oriented to person and place
Day 46	No myoclonic jerks noted Patient discharged to her local hospital for further neurorehabilitation
4 months after VNS	Obstetric decision that no additional fetal monitoring required Midwives have no specific clinical concerns about pregnancy. Occasional tremors in upper and lower limbs but they are inconsistent and can be stopped when the patient concentrates
5 months after VNS	No further seizures Continues on clonazepam and Permapanel No focal neurology Patient remains fatigued Mobilizing using Sara Steady Sit-to-stand aid Concomitant low mood Fetal ultrasound, including anomaly scan, normal Delivery plan: spontaneous vaginal delivery
6 months after VNS	Preterm premature rupture of membranes at 33 weeks gestation Transverse fetal lie and fetal bradycardia Category 1B cesarean section with spinal anesthesia to deliver baby Baby premature but otherwise healthy [ Apgar scores = 3 (1 min), 7 (5 min), 8 (10 min)] Baby floppy at birth and heart rate < 60 bpm, however, increased to >100 bpm after 5 inflation breaths within 3 min of life Baby breathing became regular after 3 min of life Oxygen saturation >95% by 5 min of life Baby pink and well-perfused, normal breath and heart sounds, soft abdomen Obstetric review concluded patient is making good operative recovery Baby remained on continuous positive airway pressure (CPAP) for 48 h after birth Enteral feed started for baby on day 5 after birth MRI head for baby—normal

#### 2.3.2. Pregnancy outcome

Except for a minor vaginal bleed lasting 2 days, pregnancy was uneventful: repeat fetal monitoring by ultrasound at day 17, 4 and 5 months after VNS implantation and a fetal anomaly scan were unremarkable and a spontaneous vaginal delivery was planned. The patient experienced preterm premature rupture of membranes (PPROM) at 33 + 2 weeks gestation, and went into preterm labor with transverse fetal lie and fetal bradycardia. The baby was delivered via emergency cesarean section under spinal anesthesia, and admitted to the neonatal ICU due to prematurity, but was discharged without neonatal concerns. Our patient made an unremarkable obstetric recovery.

## 3. Discussion

We present a case of a 5-weeks pregnant 30-year old female who presented with NORSE. Etiology remained unresolved, although drug overdose leading to subtle hypoperfusion was considered. VNS implantation on the 26th day after presentation was followed by reduction and eventually resolution of seizure activity.

This case highlights the safety and effectiveness of VNS use in pregnant patients presenting with super-refractory status epilepticus, and specifically, NORSE. Ultimately, our experience suggests that VNS implantation can be considered early in pregnancy to facilitate seizure cessation and reduce the need for multiple ASMs, many of which are teratogenic, especially when administered together.

The use of VNS in pregnancy, and the corresponding obstetric and fetal outcomes are crucial to study, however, it is important to note that in all reported studies except one, VNS was implanted before conception. Moreover, in all studies, VNS was used to control long-standing epilepsy or reduce the need for ASM polytherapy. The acute implantation of VNS in pregnancy (specifically the first trimester) to control NORSE or super-refractory status epilepticus has not previously been reported. One study found that although the rate of obstetric interventions was higher than the average pregnant population, there was no VNS-related teratogenicity in the fetus (9), with normal mean Apgar scores and birth weights for the infants. Only 1/26 infants had a major malformation: the mother was managed with four ASMs, suggesting that VNS could potentially reduce the number of ASMs used and thus contribute to reducing the teratogenicity associated with ASM polytherapy. In another study following five pregnancies, four outcomes were positive, and one ended in a spontaneous abortion (10). No teratogenicity or VNS-related complications were observed during pregnancy or delivery. A further study followed a patient in whom VNS was implanted 2 months before conception, and decreased her seizure frequency significantly (11). Lastly, in a case series, three out of four patients had obstetric complications needing cesarean sections. Six out of seven babies were healthy; one had intellectual disabilities (12). Only, one case report outlines a patient in whom VNS was implanted during the 3rd trimester of pregnancy (13). The device was activated immediately without any complications and drastically improved seizure control. The patient delivered a healthy baby at 37 weeks' gestation via a cesarean section. Two overarching conclusions can be suggested from these cases—fetal outcomes are generally positive, however, obstetric complications might be increased in pregnant patients with VNS: the latter can be multifactorial, with epilepsy being a significant factor and spontaneous abortion and prepartum complications being strongly linked to ASM polytherapy. Our patient did not experience any obstetric complications after VNS implantation before delivery, and delivered a premature but otherwise healthy baby via a cesarean section in keeping with the literature reviewed.

The mechanism by which VNS controls seizure activity is relatively unknown. Uncertainty surrounding VNS function might raise concerns about its safe use in pregnancy, especially considering its apparent effects on neuroendocrine functions, and its influence on the ovaries and uterus. VNS can activate the hypothalamus, which is a key structure in the hypothalamic-pituitary-gonadal axis (14). However, while ascending fibers of the vagus nerve can activate the hypothalamus, they do not have an apparent effect on the downstream target organs (15).

Whilst contradictory evidence exists as to whether the vagus nerve can directly influence pelvic organ function (15, 16), other studies suggest it has little impact on pregnancy (14).

A further concern could arise due to a possible malfunctioning of the device during pregnancy, however one case demonstrated that suboptimal functioning during organogenesis period did not lead to any morphological abnormalities in the fetus (17), while another study observed no malfunction of VNS during pregnancy or after birth (18). Our patient did not experience any VNS malfunctioning from implantation to discharge to her local hospital, and until she delivered her baby.

A final safety concern includes the rapid rate at which VNS is titrated up in NORSE. In our patient, the VNS current was increased from 0.25 to 1.25 mA within 7 days of implantation, an increase which would otherwise be spread out across several weeks. This did not negatively impact the patient or the fetus. Stimulus-related side-effects such as coughing, hoarseness and throat pain were not reported. The fast titration of VNS has been documented in other case reports outlining the use of VNS in NORSE (4–8), however, it had not been reported in pregnant patients presenting with NORSE.

Due to the severity of the patient's condition, multiple interventions were trialed in a short timeframe, including introduction of a ketogenic diet, Brivaracetam, Anakinra, and plasmapheresis. There is a possibility that one or more of these measures could have contributed to seizure termination. However, the following evidence supports VNS treatment being the main contributor to status cessation:

Adequate ketosis requires sustained blood ketone levels above 0.5 mmol/L. Our patient's blood ketones remained consistently under 0.5 mmol/L, except for one instance where they reached 1.4 mmol/L, confirming that adequate ketosis was not achieved, which reduces the likelihood of seizure termination due to a ketogenic diet. Furthermore, the patient's myoclonus did not recur when the ketogenic diet was stopped.

Brivaracetam is unlikely to have caused seizure cessation; one review investigating the use of Brivaracetam in SE found that the longest time to seizure termination with Brivaracetam was 24 h, and < 50% of SE patients responded to Brivaracetam (19). Although, Anakinra and plasmapheresis have been successful in managing NORSE, any therapeutic effects would have become apparent in the first 7 days after initiation.

Our patient experienced a nearly immediate and sustained positive response when VNS was switched on; this also allowed ketamine weaning to be commenced, which had not previously been possible with any other therapeutic interventions.

Another point to address is the apparent improvement noted on EEG on day 19. This could be attributed to the introduction of Ketamine; however, the patient remained in intractable SE despite this, indicating that Ketamine alone could not provide seizure freedom.

Our case-report and subsequent literature-search suggest that acute VNS implantation is a safe and effective therapeutic option in pregnant patients presenting with NORSE. The patient experienced notable neurological recovery without significant or long-term obstetric or fetal compromise. Our findings are especially important because they highlight how VNS might significantly reduce seizure activity and increase favorable outcomes in a scenario which otherwise carries a poor prognosis.

## Data availability statement

The datasets presented in this article are not readily available because of ethical and privacy restrictions. Requests to access the datasets should be directed to the corresponding author.

## Ethics statement

Ethical review and approval was not required for the study on human participants in accordance with the local legislation and institutional requirements. The patients/participants provided their written informed consent to participate in this study. Written informed consent was obtained for the publication of this case report.

## Author contributions

MJ: conceptualization, writing—original draft, writing—reviewing and editing, visualization, investigation, and methodology. LD and JW: investigation and writing—reviewing and editing. RG, SB, MA-C, and RS: investigation. LM: conceptualization, investigation, methodology, writing—reviewing and editing, and supervision. All authors contributed to the article and approved the submitted version.
