# Supplementary material for: Safe and effective implantation and use of vagal nerve stimulation in new-onset refractory status epilepticus in early pregnancy: a case report
Source: Front Neurol. 2023 May 15;14:1183080. doi: 10.3389/fneur.2023.1183080 (PMC10225632; doi:10.3389/fneur.2023.1183080)
Supplement: Supplementary file 1 [file Data_Sheet_1.pdf]

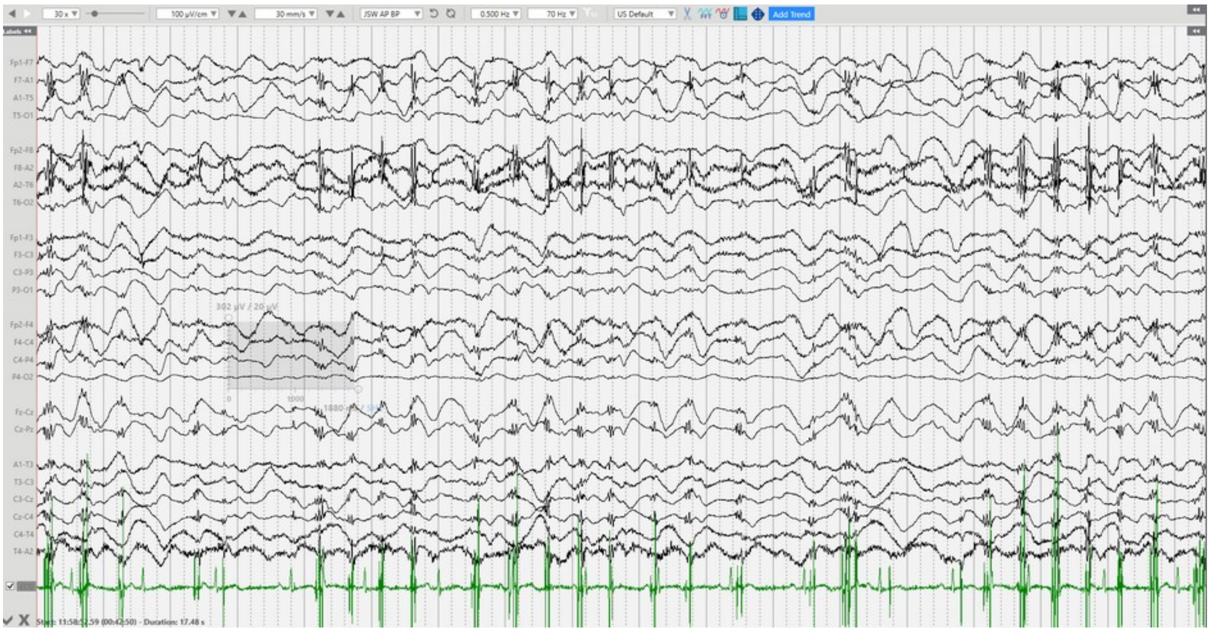

Supplementary Figure 1 (a-e): Sequential EEG Excerpts (*bipolar montage*). (a) *Long runs of jerks with slow and spikes on EEG seemingly concurrent with myogenic activity (7 days from onset).*

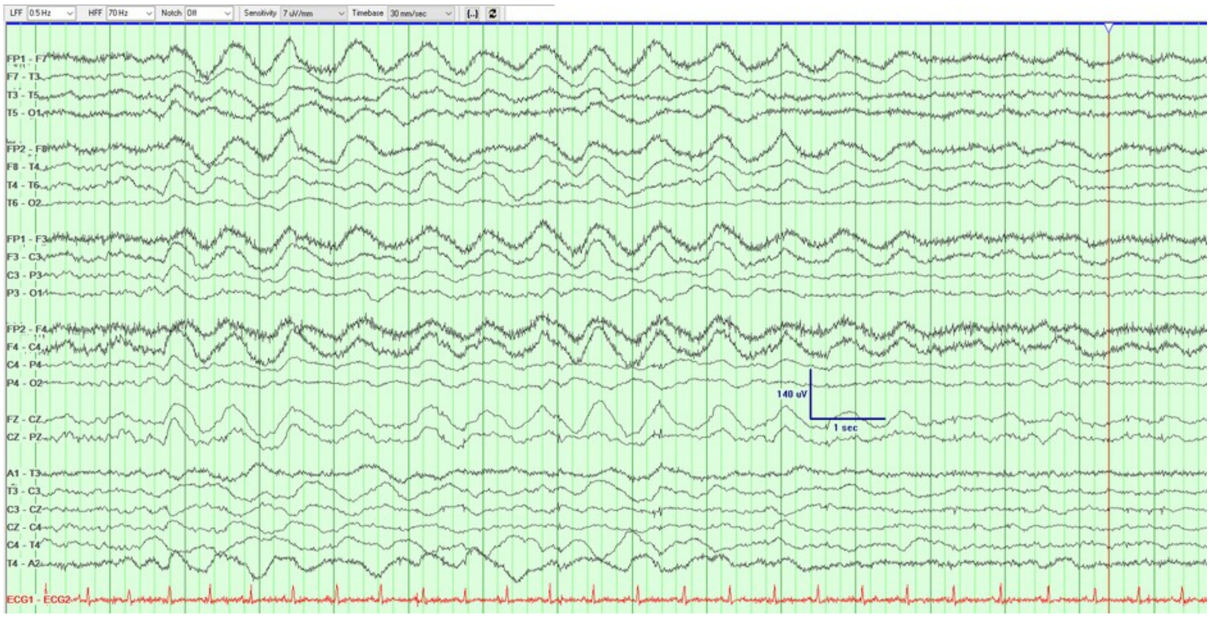

(b) *EEG at rest (19 days from onset) showing bifrontal rhythmic slowing .*

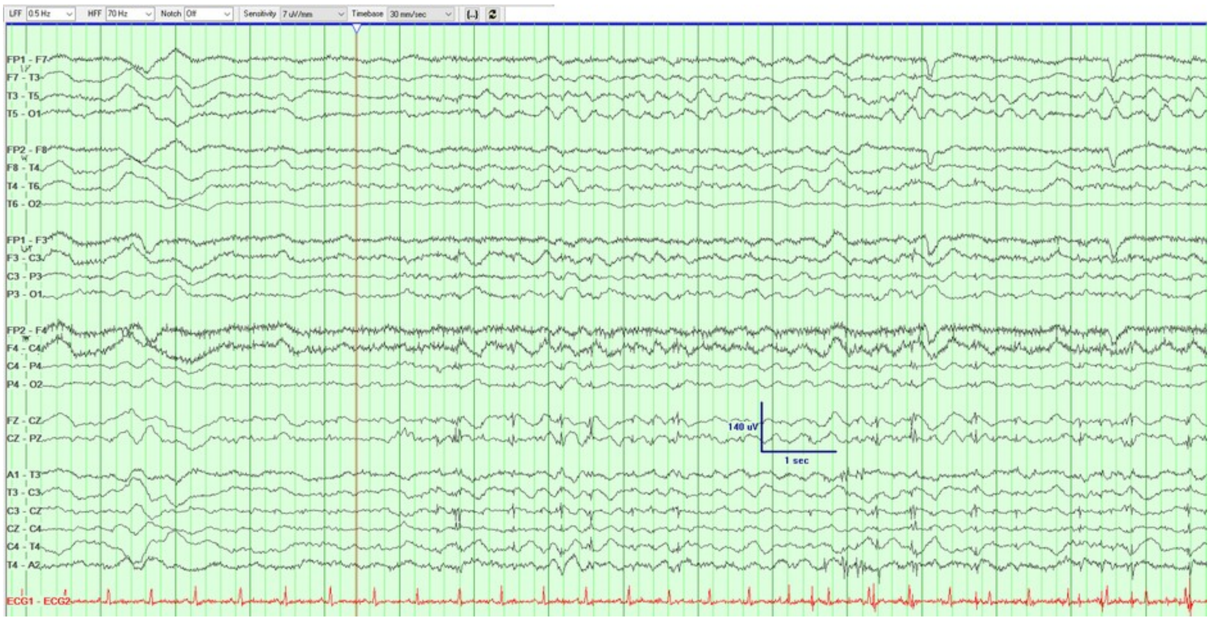

(c) *EEG with stimulation (correlating with vertical red bar) on day 19 from onset showing low amplitude midline spikes and EEG background change after stimulation.*

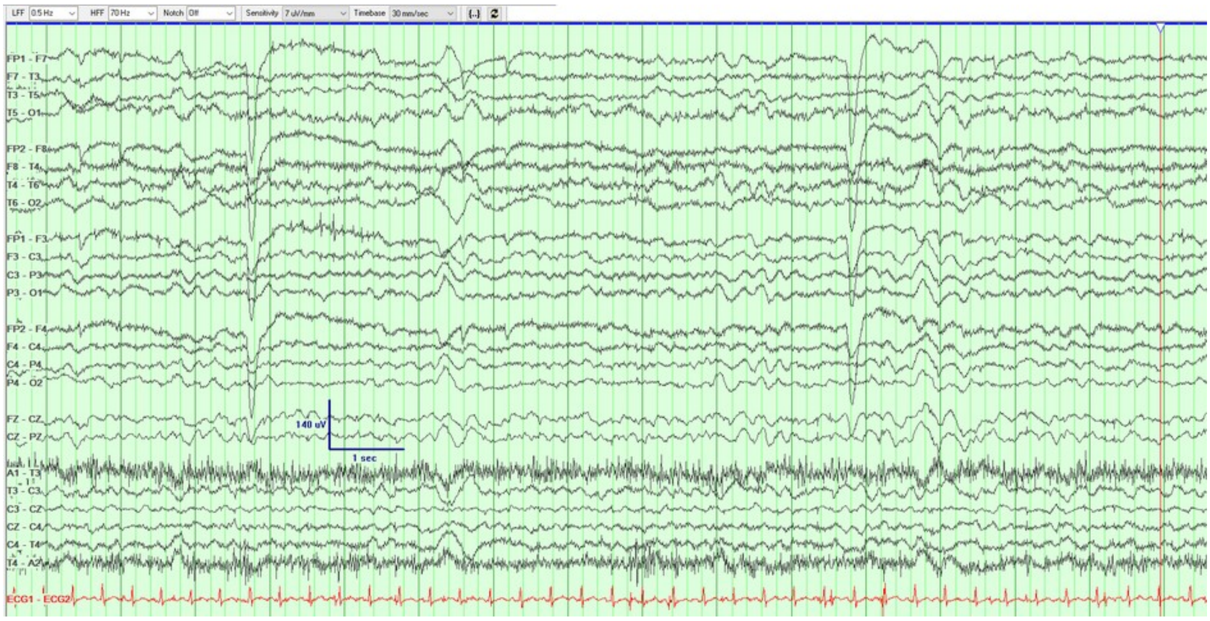

(d) *EEG at rest (28 days from onset) showing a wider mix of activities than previous studies. Ongoing spikes and multifocal myoclonus with stimulus-sensitivity were still present.*

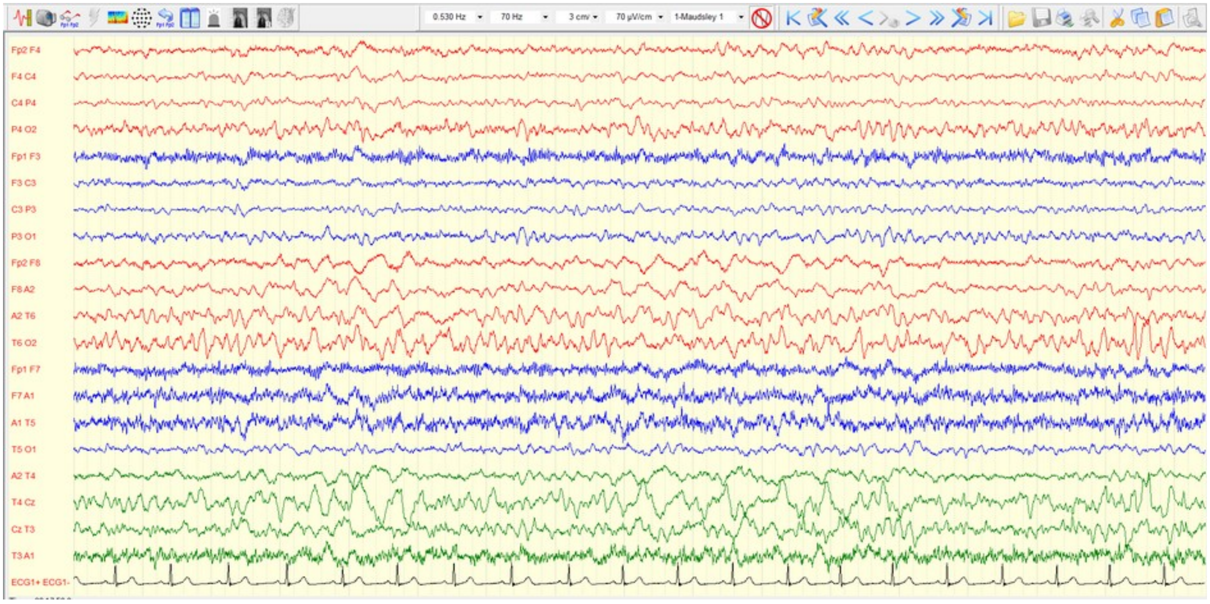

(e) *EEG at rest (5 months from onset) showing a wide mix of activities. Myoclonic jerks were no longer present.*
